# At Home—Somewhere in France

**Published:** 1919-12-20

**Authors:** 


### CHRISTMAS CONTRASTS.

### At Home—Somewhere in France.

Christmas morning in England, 5 a.m. The lights in their coloured shades are half-switched on, washing-basins are passed quietly round, bulky parcels loom on each locker, tired women look up from their beds with a half shame-faced interest. It is a long time since Santa Claus last visited them. An excited rustle of paper comes from a youngster's bed, and then the hoot of a tin trumpet, which is firmly suppressed by the nearest nurse.

"No talking till six o'clock, Polly—you mustn't blow your squeaker yet."

There are big fires in the grates, radiators in the bathrooms and cold corners, and throughout the ward there is a sense of warmth and comfort, and good cheer.

### In the Great War.

Do you remember Christmas a year or two ago? Night sister stumbled down the cinder-path and rapped on our tents at 5 a.m. It was freezing hard—dark but clear, and the wind that always blows before the dawn swept down the hill and shook the stiffened tent flaps.

When you sleep in a tent in winter you divest yourself of many queer garments before you begin to dress—and then you dress quickly. Snow boots over your shoes until your toes thaw, a woolly golf coat, overcoat, muffler, lined gloves, and a clean muslin cap—and away up the cinder-path to the Church Tent and six o'clock Communion. The Sanitary Squad clanked by on the way up, and two chilly batmen were lighting the Soyer's stoves outside the mess hut.

Inside the Church Tent, warmed by two most imperfect "Perfection" oil-stoves, was a small crowd of patients, orderlies, sisters, and doctors, kneeling on folded blankets on the uneven tarpaulin, each one alone with his Maker and his thoughts. The electric wires had fused, and candles made islands of light about the altar and the harmonium. Towards the end of the service we sang a hymn, and from the tented wards round about a hundred squeakers, tin trumpets, and mouth-organs joined the chorus "Hark, the herald angels sing," and the joyous voices calling from bed to bed made not an interruption but an addition to our praise.

Outside once more, stamping our feet and hands in the frosty air, we exchanged greetings, looked into the nearest tents to call "A Happy Christmas" to the men, and to laugh at the harassed night staff who were trying to wash, feed, dose, and tidy fifty riotous overgrown babies in each tent. An annexe of five hundred Australians suffering from mumps and high spirits is no light charge for the most energetic night sister. A few weeks earlier the authorities arranged that all patients on full diet should have goose for their Christmas dinner, except the Australians, who, with their swollen faces and sore jaws, could eat nothing better than mince; but Australia is not easily beaten, and the annexe sent word that if it could not eat goose it would prefer to die in the attempt. That won the day.

After breakfast, doctors, nursing staff, and orderlies worked hard to finish dressings and treatment in good time, and leave the patients free. A frosty sun turned the tents from silver to pale gold and lit a gleam on the sea two miles away across the marshes. At eleven o'clock the Church Tent was crowded to its utmost capacity, and many a man sang the Christmas hymns as he had not sung them since his childhood.

Then came the great event of the day. Christmas dinner. Long before the bugle called "Come to the cookhouse door, boys," a queue of orderlies and patients had lined up, each man carrying a shining tin food-heater, a soup bucket, or bread tray, and now the sergeant cook and his men distributed the fruit of their labours—a first-class meal. A well-run cookhouse goes a long way towards making a happy hospital. The Colonel, the matron, the registrar, and sergeant-major went through every ward, saw that the dinner was good and plentiful, and gave and received many good wishes. In the acute wards there were some who could not manage a heavy meal, and for these orderlies and sisters cooked queer little dishes. Smith, with the double amputation, guessed he would like a bit of toasted cheese, done on a tin plate over the oil stove; and little Ginger from Bermondsey, Army age 19½, real age 16 years, in whose intestine eleven holes were sewn up last week, wanted a soft fried egg, a cup of bovril, and, if possible, some pink jelly afterwards. In the annexe at the top of the camp a visitor might have seen rows of determined men sitting up in bed among grey blankets, each swollen face swathed in bandages, each button mouth gnawing at a goose's drumstick, over which each pair of far-seeing steel-blue eyes proclaimed the fact that its owner had never been beaten yet.

Letters. It is an inconsequent bugle-call, but it brings a stampede to the post corporal's tent. He stood on a box outside and called the names of wards or inmates, regimental numbers, and units. For the lucky ones it was the crowning moment of the day. To be alone with your treasure is the need, alone among the crowd. By a kind of freemasonry they turn away from each other, turn inward to their own solitude, draw a magic circle across which no other will intrude until the spell is broken. If you watch quietly you will see. Smith is back in "Braadford," listening to the whirr of the looms and to the music of his people's tongue; Johnson is in Dingbat Alley, Sydney; and "Canada's" thoughts are in a cleaner, greener land. But the latest comers are unlucky. All their mail has gone up to their units, and may follow them round for months. That is the drawback of coming down

B

**Christmas Contrasts**—(*continued*)

wounded or sick just before Christmas. After Christmas a "cushy" wound is a stroke of luck, and properly managed may keep a man out of the line until the bad weather is over.

Somebody could play the fiddle, and each ward waited eagerly for its turn, filling in the time with songs, clog dancing, the gramophone; and one "Aussie"—a well-known actor from Melbourne, they said—gave some very good monologues: "Spotty"; "Romeo and Juliet," from "The Sentimental Bloke"; and an account of two "Diggers" on leave in London for the first time—"Moving Stairways?" "Don't show yer ignorance, Bill. We've got heaps of those in Melbourne, Miss."

The time passed very quickly. Tea was over—"some" tea, with girdle cakes cooked on the coke-stove top, tinned-crab sandwiches, nuts, and trifle made in washing-basins and reinforced with a little ward brandy. The helpless patients were washed, and their wounds dressed again. More songs followed, and supper. Night closed in dark and cold. A muffled murmur and the yellow flicker across the sky over the hills warned us that there was no peace up the line. Tired patients settled down to sleep. The night staff came on duty, and the day staff went over to the quarters, eager for supper and the chance to read their own letters before "lights out." Lamps twinkled from the bell tents, and huge, distorted shadows moved on the canvas. Over by the mess hut a crowd jostled round the Soyer's stoves. Water had been short for days by reason of burst pipes and frozen taps, and the allowance was half a canful each—to wash in and boil up again for a hot-water bottle. With care you could wash in it once more in the morning.

The camp was soon asleep. The white tent tops shone dimly on the hillside, and Orion shouldered his way across the dark sky above. Time passed. Then a faint bugle sounded, another nearer, and then our own—"Fall in," "At the double." A convoy. The hurrying footfall of stretcher-bearers passed up the path, and scraps of conversation drifted over to the bell tents: "A hundred and fifty walkers, fifty stretchers." "Yes, heavy fighting this morning." And then below, on the camp road, the faint sound of ambulances drew nearer. It was 1 A.M. Christmas-day was over.

